# Identification and evaluation of important agronomic traits in 49 synthetic hexaploid wheat (AABBDD) germplasm resources grown on the Qinghai-Tibet Plateau, China

**DOI:** 10.1270/jsbbs.25015

**Published:** 2025-11-13

**Authors:** Shuxiang Yin, Jicheng Shen, Fahui Ye, Xia Li, Caixia Zhao, Chaobo Liu, Meixi Song, Qingxu Wang, Demei Liu, Ruijuan Liu, Shunzong Ning, Lianquan Zhang, Huaigang Zhang, Yuhu Shen, Wenjie Chen

**Affiliations:** 1 Qinghai Provincial Key Laboratory of Crop Molecular Breeding, Northwest Institute of Plateau Biology, Chinese Academy of Sciences, Xining, Qinghai, 81000, China; 2 State Key Laboratory of Crop Gene Exploration and Utilization in Southwest China, Sichuan Agricultural University, Wenjiang, Chengdu 611130, China; 3 Triticeae Research Institute, Sichuan Agricultural University, Wenjiang, Chengdu 611130, China; 4 Laboratory for Research and Utilization of Qinghai Tibet Plateau Germplasm Resources, Xining, 810008, Qinghai, China; 5 Key Laboratory of Adaptation and Evolution of Plateau Biota, Northwest Institute of Plateau Biology, Chinese Academy of Sciences, Xining, Qinghai, 810008, China; 6 University of Chinese Academy of Sciences, Beijing, 100049, China

**Keywords:** synthetic hexaploid wheat, agronomic traits, germplasm resources, genetic diversity, Qinghai-Tibet Plateau

## Abstract

In this study, 49 ABD-type synthetic hexaploid wheat germplasm resources grown on the Qinghai-Tibet Plateau in 2022–2024 were measured for important agronomic traits. Clustering and principal component analyses were applied to classify and compare relevant indicators for a comprehensive evaluation. The aim was to identify outstanding synthetic hexaploid wheat germplasm and establish a new germplasm resource foundation for the breeding and improvement of new wheat varieties in the Qinghai-Tibet Plateau and other regions with similar climatic conditions. The coefficient of variation for important agronomic traits ranged from 1.63% to 22.02%, with the highest coefficients of variation observed for spike stem node length, plant height, and thousand-grain weight. Cluster analysis revealed that the 49 synthetic hexaploid wheat germplasm resources could be divided into eight major clusters. Among them, the quality traits of the accessions belonging to clusters Ⅴ exhibited excellent performance, while the accessions with outstanding yield-related traits were concentrated in clusters Ⅲ and Ⅷ. Using the gray relational analysis method for comprehensive evaluation, the five germplasm resources with the highest scores were ABD-SHW-15 (64.53), ABD-SHW-6 (64.39), ABD-SHW-5 (63.70), ABD-SHW-14 (63.18), and ABD-SHW-38 (62.33).

## Introduction

Wheat is a highly adaptable and widely distributed staple crop worldwide (Cheng *et al.* 2024, He *et al.* 2011, Li 2010), serving as the primary food source for 35% to 40% of the global population. Its high and stable yield is of great significance in safeguarding global food security. Wheat (*Triticum aestivum* L., AABBDD, 2n = 6x = 42) was formed by undergoing two heterologous polyploidizations. The first occurred about 300,000–500,000 years ago, when *Triticum urartu* Tum. ex Gandilyan (AA, 2n = 2x = 14) and *Aegilops speltoides* Tausch (BB, 2n = 2x = 14), or its close relatives (B genome donors), underwent natural hybridization and natural doubling of chromosomes to form the early-cultivated *Triticum dicoccoides* Koern. et Schweinf. (AABB, 2n = 4x = 28). The second heterologous polyploidization occurred about 8000 years ago, when *Triticum turgidum* L. was crossed with *Aegilops tauschii* Coss (DD, 2n = 2x = 14), with a natural doubling of chromosomes, resulting in the formation of wheat (Dvořák *et al.* 1993, Kihara 1944, Kilian *et al.* 2007, Petersen *et al.* 2006). Due to the involvement of only a few related species in this process, its genetic base is relatively narrow. Furthermore, during long-term domestication, breeders have focused on achieving high yields, using extensive intervarietal hybridization and relying heavily on a few elite parents to develop new cultivars. While this approach has ensured the selection of excellent agronomic traits to a certain extent, it has also resulted in a convergence of morphological and genetic traits among many modern cultivated cultivars, thereby reducing genetic diversity. These factors have collectively contributed to the narrow genetic base of modern cultivated wheat cultivars (Song *et al.* 2022), which is manifested as stagnant yield increases, difficulties in improving quality, and decreased or lost resistance to adverse environments and pests (Song *et al.* 2022). Therefore, further enhancing the genetic diversity of wheat germplasm resources has become an essential prerequisite for developing wheat cultivars with key traits such as high yield, excellent quality, and stress resistance. Studies have shown that related species of wheat harbor abundant genetic resources. *Triticum turgidum*, the donor of the A and B genomes of common wheat, possesses numerous favorable traits, such as drought tolerance, tolerance to poor soil conditions, disease resistance, high protein content, high nutritional value, and good processing qualities. *Aegilops tauschii*, the donor of the D genome of wheat, has accumulated numerous beneficial genes (Gaurav *et al.* 2022) for grain quality (Bo *et al.* 2022, Mohamed *et al.* 2022), disease resistance (Kou *et al.* 2023, Marais *et al.* 1994, Nakano *et al.* 2015), drought resistance (Mokhtari *et al.* 2024), heat resistance (Wright *et al.* 2024), and other traits during evolution. It exhibits rich genetic diversity and functional adaptability in agronomic traits such as stems, leaves, spikes, and seeds (Song *et al.* 2022). Relevant research indicates that, compared to common wheat, synthetic hexaploid wheat (SHW) (Wan *et al.* 2022, Zhang *et al.* 2007), which was obtained through hybridization between *Triticum turgidum* and *Aegilops tauschii* followed by chromosome doubling, contains rich genetic resources from wild relatives and exhibits higher genetic diversity (Bhatta *et al.* 2019). This diversified genetic resource enables SHW to frequently exhibit traits such as drought tolerance, salt-alkali tolerance, and cold tolerance, making it better suited to extreme climatic conditions. This characteristic is highly beneficial for agricultural production in plateau, arid, and saline-alkali regions. Wan *et al.* (2023) proposed the “large population with limited backcrossing” method to improve SHW and developed the new wheat cultivar “Chuanmai 42”, which achieved a 35% higher yield compared to contemporary regional trial control cultivars and set a new yield record for commercial wheat cultivars in the southwestern wheat region. Del Blanco *et al.* (2001) found that in six backcross populations between selected SHW and spring wheat lines, over 80% of the lines exhibited significantly higher thousand-grain weights than their respective backcross parents, suggesting that utilizing the higher thousand-grain weight advantage of SHW could be one of the most effective approaches for modern wheat improvement. Hao *et al.* (2019) reported that among the QTL loci for 86 yield-related traits detected in 14 environments in Sichuan province, China using a recombinant inbred line population derived from SHW-L1 and Chuanmai 32, 40 favorable alleles originated from SHW-L1. Thus, employing SHW as a genetic bridge in breeding programs is an effective strategy for wheat genetic improvement.

Qinghai Province is situated in the transitional zone between the Qinghai-Tibet Plateau and the northwest inland region of China, characterized by a typical cold continental climate and a unique ecological environment. In recent years, wheat production in this region has faced several challenges, including stagnating yields, degradation of existing cultivars, a severe shortage of genetic diversity in elite lines, and increasing genetic homogeneity among wheat varieties. These issues are further exacerbated by harsh environmental stresses such as low temperatures and drought, as well as limited progress in breeding technologies. Despite the recognized potential of SHW as a genetic bridge to broaden the genetic base of common wheat, its application in wheat improvement programs in the Qinghai-Tibet Plateau remains limited. In this context, the identification, evaluation, and utilization of SHW germplasm hold great significance for enriching the genetic diversity of wheat cultivars and accelerating the development of new varieties with improved yield, stress tolerance, and excellent quality adapted to this challenging environment.

In this study, we continuously assessed the agronomic traits of 49 previously synthesized ABD-type SHW germplasm resources over the three-year period in the eastern Qinghai-Tibet Plateau and conducted a comprehensive evaluation using gray relational analysis. The objective was to identify SHW germplasm resources with superior agronomic traits suitable for cultivation in this region, thereby broadening the genetic base of wheat and providing novel genetic resources for wheat improvement in the Qinghai-Tibet Plateau and other high-altitude regions with similar environmental conditions.

## Materials and Methods

### Experimental materials

**Fig. 1.**
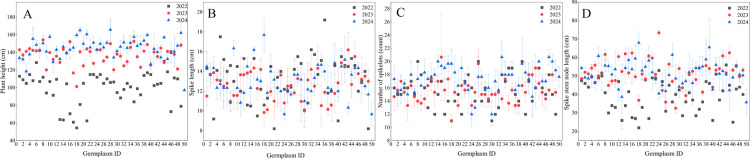
Changes in plant phenotypic of plants in 2022–2044.

**Fig. 2.**
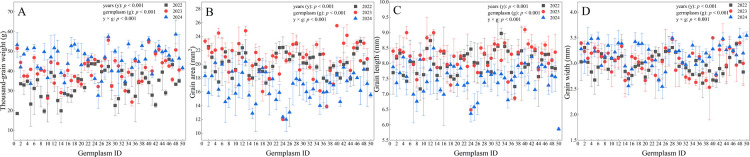
Changes in grain characteristics of plants in 2022–2044.

**Fig. 3.**
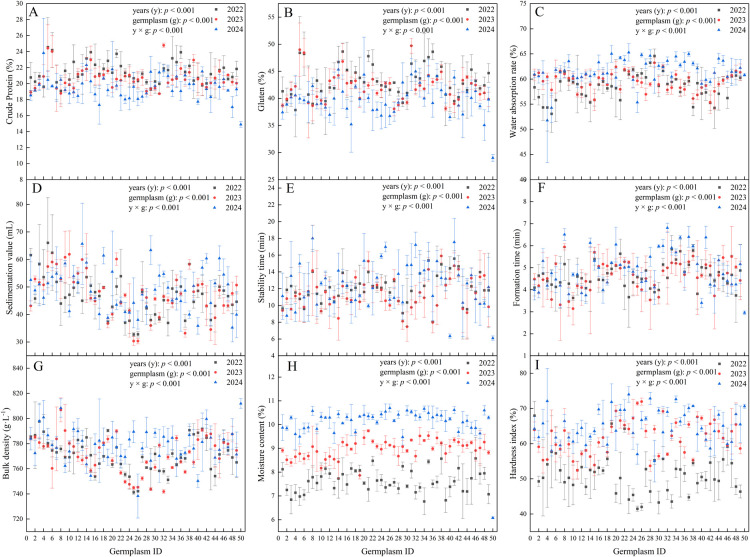
Changes in grain quality of plants in 2022–2044.

The 49 SHW germplasm resources used in this study were obtained through crosses between 30 *Triticum turgidum* and 15 *Aegilops tauschii* (Table 1), and were provided by Sichuan Agricultural University. The control material was Gaoyuan 448, a widely cultivated spring wheat cultivar in Qinghai. All genetic materials were maintained at the Qinghai Provincial Key Laboratory of Crop Molecular Breeding. In Figs. 1–3, the germplasm ID 50 corresponds to the control cultivar Gaoyuan 448.

### Experimental design

This study was conducted over three consecutive years from 2022 to 2024 at the Haidong Eco-Agriculture Experimental station (102°19ʹ32ʺE, 36°28ʹ60ʺN) of the Northwest Institute of Plateau Biology, Chinese Academy of Sciences. The area is a typical transition mosaic from the Loess Plateau to the Qinghai-Tibet Plateau, with an elevation of 2016 m, average annual temperature of 3.2–8.6°C, average annual rainfall of 319.2–531.9 mm, evaporation rate of 1275.6–1861 mm, average duration of annual sunshine of 2708–3636 hours and frost-free period of about 90 days. The experiment was conducted in a two-factor randomized group design. Sixty grains of each material were planted in three rows on medium-fertile land. The rows were 2 m long and 0.2 m apart. Field management strategies such as fertilization, weeding, irrigation, and pest control were the same as in conventional breeding fields. When the test material reached maturity, it was promptly harvested by hand, and the seeds were dried in the sun and stored in a suitable, ventilated place.

### Determination of phenotypic traits

After harvest, 3 intact plants were randomly selected from each material type. Plant height (PH), spike length (SL), and spike neck node length (SNL) were measured with a tape measure and a scale,while the number of spikelets (SN) was determined by manually counting the spikelets on each spike.

After grain threshing, 200 grains from each germplasm were randomly selected to measure the grain length (GL), grain width (GW), and grain area (GA) using a Marvin analysis grain photoelectric analyzer with five repetitions. From each sample, seeds were randomly selected and weighed with an electronic balance of an accuracy of one thousandth, and the measurement was converted to thousand-grain weight (TGW).

### Determination of grain quality

After grain harvest, broken wheat, stones, and other impurities were removed from the germplasm resources. A grain analyzer (Model: MARVIN-U) was used to measure grain quality-related traits, including moisture content (MC), crude protein (CP), gluten, water absorption rate (WA), sedimentation value (SED), hardness index (HI), bulk density (BD), stability time (ST), and formation time (FT). All indices were measured three times.

### Gray relational analysis

In this study, the 17 agronomic traits of the 49 SHW germplasm resources were correlated to varying extents, complicating the accurate assessment of the overall performance of the germplasm. Gray system theory facilitates the standardization of indicators with different dimensions, types, and physical meanings, allowing for a more accurate representation of the true performance of the evaluated materials. Moreover, within the comprehensive evaluation framework, the weighting of each indicator reduces the impact of subjective factors. This method is straightforward and effective in practical applications, as it not only identifies the excellent traits of the materials but also reveals potential deficiencies, providing a more comprehensive and accurate assessment. According to gray relational theory, the tested materials are considered part of a gray system, with each material representing a factor in the system. Indices of the optimal material are used as the reference sequence *X_0_*. Seventeen agronomic traits, such as plant height, spike length, spike stem node length, and crude protein content, are designated as comparative sequences *X_1_* through *X_14_*. The degree of similarity between each comparative sequence (*X_i_*) and the reference sequence (*X_0_*) determines both the gray relational coefficient and the gray relational degree.

According to the method described by Zhao *et al.* (2023), the correlation coefficient was calculated using the following formula:


$$A_{k}={\text{min}}_{i}{\text{min}}_{k}\left|X_{0}(k)-X_{i}(k)\right|+\rho {\text{max}}_{i}{\text{max}}_{k}\left|X_{0}(k)-X_{i}(k)\right|$$

$$B_{k}=\left|X_{0}(k)-X_{i}(k)\right|+\rho {\text{max}}_{i}{\text{max}}_{k}\left|X_{0}(K)-X_{i}(k)\right|$$

$$\Psi_{i}(k)=\frac{A_{k}}{B_{k}}$$

In the formula: *Ψ_i_*(*k*) represents the relational coefficient between the comparative sequence Xi and the reference sequence *X_0_* at point *k*. $\left|X_{0}(k)-X_{i}(k)\right|$ represents the absolute difference between the comparative sequence *X_i_* and the reference sequence *X_0_* at the *k-th* point. ${\text{min}}_{i}{\text{min}}_{k}\left|X_{0}(k)-X_{i}(k)\right|$ represent the minimum and maximum absolute differences across all sequences and all data points, respectively. ${\text{max}}_{i}{\text{max}}_{k}\left|X_{0}(K)-X_{i}(k)\right|$ represent the maximum and maximum absolute differences across all sequences and all data points, respectively. *ρ* is the resolution coefficient, ranging from 0 to 1. In this study, it was set to 0.5, a commonly used value that balances sensitivity and stability.

Weighted correlation formula:


$$\gamma_{i}=\sum_{k=1}^{n}W_{i}\times \Psi_{i}(k)$$

In the formula, $W_{i}$ represents the weight of each indicator, determined according to the method described in the literature. $\gamma_{i}$ represents the weighted association degree.

### Statistical analysis

The phenotypic data were summarized using Microsoft Excel and plotted using sigmaplot12.5. Significance analysis, correlation analysis, principal component analysis, and cluster analysis (Euclidean distance class average method) of plant phenotypic morphology, grain phenotypic morphology, and grain quality were performed using SPSS 21.0 software.

## Results

### Plant phenotypic and grain characteristics and grain quality

From 2022 to 2024, the plant height of the 49 SHW germplasm resources showed a consistent upward trend, with average values increasing from 99.82 cm to 145.78 cm, representing a 46.04% overall increase. Concurrently, the coefficient of variation declined from 18.08% to 7.00% (Table 2). Notably, all germplasm resources, except ABD-SHW-44, had their lowest plant height in 2022 (Fig. 1A). The three-year mean plant height (127.41 cm) was significantly higher than that of the local variety Gaoyuan 448, and all SHW lines exceeded its height (Table 3).

The spike length ranged from 8.20 cm to 19.20 cm, with relatively stable average values across the three years. The coefficient of variation declined over time (Table 2). Among the materials tested, the spike length of ABD-SHW-3, ABD-SHW-13, ABD-SHW-16, ABD-SHW-21, ABD-SHW-34, and ABD-SHW-46 increased annually (Fig. 1B). On average over the three years, the spike length of synthetic hexaploid wheat was 3.33 cm longer than that of Gaoyuan 448. Additionally, the average spike length of all SHW lines tested exceeded that of Gaoyuan 448 (Table 3).

The number of spikelets per spike ranged from 11.00 to 20.67, with average values increasing annually from 15.45 to 17.24, representing an overall increase of 11.58% from 2022 to 2024. Meanwhile, the coefficient of variation decreased slightly (Table 2). Over the three years, the average number of spikelets was 16.10, which was 1.57 fewer than that of Gaoyuan 448, but the average number of spikelets for five of the tested SHW lines was higher than that of Gaoyuan 448 (Table 3).

The spike stem node length ranged from 22.00 cm to 73.37 cm, with average values increasing annually from 39.53 cm to 51.65 cm, representing a 30.67% increase from 2022 to 2024. The coefficient of variation decreased over time (Table 2). The three-year mean was 47.60 cm, which was 11.84 cm longer than that of Gaoyuan 448, and all SHW lines exceeded the local variety (Table 3).

From 2022 to 2024, the thousand-grain weight of the 49 SHW germplasm resources ranged from 18.53 g to 58.60 g. The average values increased annually and were 33.23 g, 40.48 g, and 45.94 g in 2022, 2023, and 2024, respectively, representing a 38.24% increase over the three-year period. Meanwhile, the coefficient of variation decreased steadily from 21.33% to 14.94% (Table 2). The three-year average was 39.88 g, slightly lower than that of Gaoyuan 448. The average thousand-grain weight of 13 tested SHW germplasm resources was higher than that of Gaoyuan 448 (Table 3).

The grain area ranged from 12.00 mm^2^ to 25.57 mm^2^. The average values for the three years were 20.16 mm^2^, 20.64 mm^2^, and 16.81 mm^2^ in 2022, 2023, and 2024, respectively. The coefficients of variation were 8.09%, 13.08%, and 12.32% for each year (Table 2). The three-year average grain area was 19.20 mm^2^, which was 3.66 mm^2^ larger than that of Gaoyuan 448, and all SHW lines showed higher average grain area than the local check (Table 3).

Grain length ranged from 6.36 mm to 9.10 mm, with average values of 8.03 mm, 8.18 mm, and 7.53 mm in 2022, 2023, and 2024, respectively (Table 2). The three-year average grain length was 7.91 mm, which was 2.05 mm longer than that of Gaoyuan 448. All tested SHW germplasm resources exhibited greater grain length than Gaoyuan 448 (Table 3).

Grain width ranged from 2.53 mm to 3.52 mm, with average values increasing annually and measuring 2.98 mm, 3.05 mm, and 3.17 mm in 2022, 2023, and 2024, respectively. The coefficients of variation for grain width were similar across the three years (Table 2). The three-year average was 3.07 mm, which was 0.48 mm narrower than that of Gaoyuan 448, and all tested SHW lines had smaller grain width than the Gaoyuan 448 (Table 3).

The effects of germplasm resources, year of growth, and their interaction on thousand-grain weight, grain area, grain length, and grain width traits were extremely significant (Fig. 2A–2D).

From 2022 to 2024, the crude protein content of the 49 SHW germplasm resources ranged from 17.06% to 24.79%, with average values showing a decreasing trend over the three years. The coefficients of variation remained relatively stable (Table 2). The three-year average crude protein content was 20.61%, exceeding that of Gaoyuan 448 by 5.72%, and all tested SHW lines had greater crude protein content than the Gaoyuan 448 (Table 3).

The gluten content ranged from 35.05% to 49.71%, with the average value showing a decreasing trend over the three years. The coefficients of variation for gluten content were similar across the years (Table 2). The three-year average gluten content (41.75%) was notably higher than that of the control variety Gaoyuan 448 by 12.72%, and all tested SHW lines had greater gluten content than the Gaoyuan 448 (Table 3).

The water absorption rate ranged from 51.78% to 65.29%, with the average values showing a increasing trend over the three years (Table 2). Although the three-year average (59.86%) was slightly lower than that of Gaoyuan 448, 17 SHW lines surpassed the control (Table 3),

The sedimentation value ranged from 30.34 to 66.05 mL, and the average values exhibited an increasing trend over the years. The coefficients of variation remained relatively consistent across the different years (Table 2). Among the evaluated lines, ABD-SHW-5 and ABD-SHW-6 exhibited comparatively high sedimentation values, while ABD-SHW-26 had the lowest (Table 3).

The stability time ranged from 6.35 min to 18.00 min, with annual averages of 12.01 min (2022), 11.39 min (2023), and 12.58 min (2024). The coefficients of variation for stability time increased annually (Table 2), respectively. The three-year average stability time was 11.99 min, which was 5.89 min longer than that of Gaoyuan 448, and the average stability time of all tested SHW was higher than that of Gaoyuan 448 (Table 3).

The formation time ranged from 3.14 min to 6.81 min, with annual averages of 4.62 min (2022), 4.65 min (2023), and 4.96 min (2024). The coefficients of variation for formation time increased annually (Table 2). The average formation time of all tested materials was 4.74 min, which was 1.79 min longer than that of Gaoyuan 448, and the average formation time of all tested SHW was higher than that of Gaoyuan 448 (Table 3).

The bulk density ranged from 738.29 g L^–1^ to 808.38 g L^–1^, with annual averages of averages of 771.17 g L^–1^ (2022), 771.66 g L^–1^ (2023), and 778.43 g L^–1^ (2024). The coefficients of variation remained relatively consistent across the different years (Table 2). The average bulk density over the three years was 773.76 g L^–1^, which was 38.14 g L^–1^ lower than that of Gaoyuan 448, and the average bulk density of all tested SHW was lower than that of Gaoyuan 448 (Table 3).

The moisture content ranged from 6.77% to 10.70%, and the average values exhibited an increasing trend over the years. The coefficient of variation for moisture content showed a decreasing trend over the years (Table 2). The average moisture content over the three years was 8.93%, which was 2.89% higher than that of Gaoyuan 448, and the average moisture content of all tested SHW was higher than that of Gaoyuan 448 (Table 3).

The hardness index ranged from 41.48% to 73.97%, and the average values exhibited an increasing trend over the years. The coefficient of variation for hardness index showed a decreasing trend over the years (Table 2). The average hardness index over the three years was 59.37%, which was 11.22% lower than that of Gaoyuan 448, and the average hardness index of all tested SHW was lower than that of Gaoyuan 448 (Table 3).

The effects of germplasm resources, year of growth, and their interaction on all grain quality traits were all extremely significant was positively correlated (Fig. 3A–3I).

### Correlation analysis

**Fig. 4.**
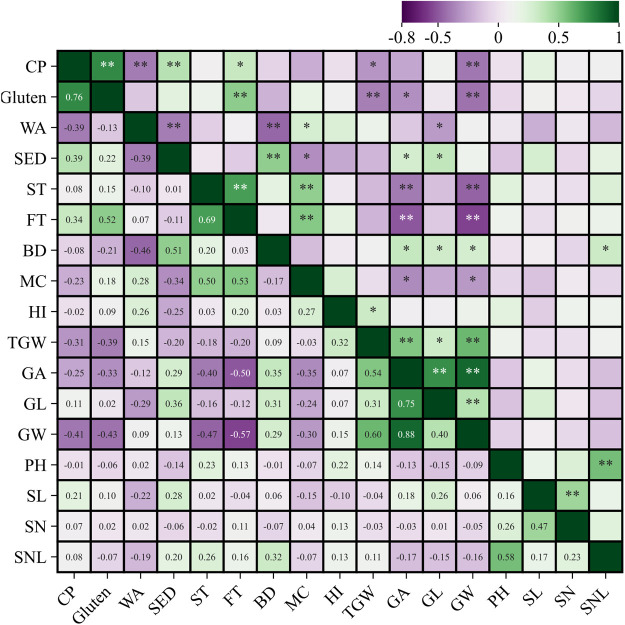
Correlation analysis between important traits in synthetic hexaploid wheat. PH, plant height (cm); SL, spike length (cm); SN, number of spikelets; SNL, spike stem node length (cm); GL, grain length (mm); GW, grain width (mm); SA, grain area (mm^2^); TGW, thousand-grain weight (g); MC, moisture content (%); CP, crude protein (%); WA, water absorption rate (%); HI, hardness index; BD, bulk density (g L^–1^); gluten (%), SED sedimentation value (mL), stability time (min), and formation time (min). * Significant differences at 5% probability level; ** significant differences at 1% probability level.

The crude protein content was highly positively correlated with gluten content and sedimentation value, as well as positively correlated with formation time. Gluten content was highly positively correlated with formation time. The water absorption rate was positively correlated with moisture content. Sedimentation value was highly positively correlated with bulk density and positively correlated with grain area, and grain length. Stability time was highly positively correlated with both dough formation time and moisture content. Formation time was also highly positively correlated with moisture content. Bulk density was highly positively correlated with grain area, grain length, grain width, and spike stem node length. The hardness index was positively correlated with thousand-grain weight. Thousand-grain weight was highly positively correlated with grain area and grain width, as well as positively correlated with grain length. Grain area was highly positively correlated with both grain length and grain width, while grain length was highly positively correlated with grain width. Plant height was highly positively correlated with spike stem node length, whereas spike length was highly positively correlated with the number of spikelets. Gluten content exhibited a highly significant negative correlation with thousand-grain weight and plant height. Water absorption rate was highly significantly negatively correlated with sedimentation value and bulk density. Stability time showed a highly significant negative correlation with grain area and grain width. Formation time also showed a highly significant negative correlation with grain area and grain width (Fig. 4).

### Cluster analysis

**Fig. 5.**
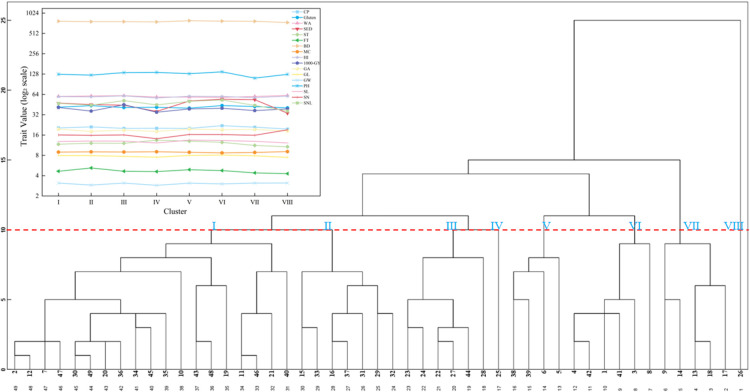
Cluster analysis of agronomic traits of 49 synthetic hexaploid wheat germplasm resources. PH, plant height (cm); SL, spike length (cm); SN, number of spikelets; SNL, spike stem node length (cm); GL, grain length (mm); GW, grain width (mm); SA, grain area (mm^2^); TGW, thousand-grain weight (g); MC, moisture content (%); CP, crude protein (%); WA, water absorption rate (%); HI, hardness index; BD, bulk density (g L^–1^); gluten (%), SED sedimentation value (mL), stability time (min), and formation time (min).

The cluster analysis of SHW test materials, performed using the Euclidean distance class average method, classified the 49 materials into eight clusters. Cluster I had the largest number of materials (nineteen materials). Cluster II included seven materials and was characterized by a longer formation time. Cluster III (six materials) exhibited a higher grain hardness index and thousand-grain weight. Cluster IV (one material) displayed a longer stability time but lower values for grain hardness index, thousand-grain weight, grain area, grain width, and number of spikelets. Cluster V (four materials) exhibited higher crude protein content, gluten content, sedimentation value, grain length, plant height, and spike stem node length but lower water absorption rate and moisture content. Cluster VI (six materials) featured higher test weight, larger grain area, longer spike length, and lower gluten content. Cluster VII (five materials) was characterized by shorter plant height. Cluster VIII (one material) showed higher grain water absorption rate, moisture content, grain width, and number of spikelets, while crude protein content, sedimentation value, stability time, formation time, test weight, grain length, spike length, and spike stem node length were relatively lower (Fig. 5).

### Principal component analysis between plant phenotypic morphology, grain phenotypic morphology, and grain quality

**Fig. 6.**
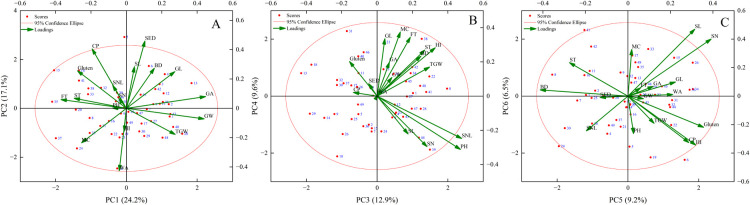
Principal component analysis of agronomic traits of 49 synthetic hexaploid wheat germplasm resources. PH, plant height (cm); SL, spike length (cm); SN, number of spikelets; SNL, spike stem node length (cm); GL, grain length (mm); GW, grain width (mm); SA, grain area (mm^2^); TGW, thousand-grain weight (g); MC, moisture content (%); CP, crude protein (%); WA, water absorption rate (%); HI, hardness index; BD, bulk density (g L^–1^); gluten (%), SED sedimentation value (mL), stability time (min), and formation time (min).

The indicators in Table 3 were standardized, and the data were subjected to principal component analysis to obtain the correlation matrix for each evaluation parameter of SHW. A total of six principal components were obtained. It can be seen that principal component 1 combined 24.19% of all information, mainly reflecting the characteristics of SHW in terms of grain width, grain area, and thousand-grain weight. Principal component 2 contributed 17.14%, mainly reflecting the characteristics of SHW in terms of sedimentation value, crude protein content, and spike length. Principal component 3 contributed 12.86%, mainly from spike stem node length, plant height, and hardness index. Principal component 4 contributed 9.60%, mainly from moisture content, formation time, and grain length. Principal component 5 contributed 9.24%, mainly from gluten content, number of spikelets, and hardness index. Principal component 6 contributed 6.48%, mainly from spike length, the number of spikelets, and moisture content (Fig. 6).

### Comprehensive evaluation

Comprehensive evaluation was conducted using the gray relational analysis method. The ratio of the eigenvalues corresponding to each principal component to the sum of the total eigenvalues of the extracted principal components was used as the weight. The top three traits with the highest weights were spike length, spikelet number, and hardness index (Table 4). The scores for the first six main components of each material and the sum of the product of the corresponding weights were used in our assessment. The top 10 lines screened using our comprehensive evaluation were ABD-SHW-15 (64.53), ABD-SHW-6 (64.39), ABD-SHW-5 (63.70), ABD-SHW-14 (63.18), ABD-SHW-38 (62.33), ABD-SHW-35 (60.02), ABD-SHW-32 (59.89), ABD-SHW-21 (59.28), ABD-SHW-20 (58.75), and ABD-SHW-36 (58.50) (Table 5).

## Discussion

The rich genetic diversity of germplasm resources is a prerequisite for variety breeding, and the coefficient of variation serves as an indicator of trait diversity (Joshi *et al.* 2023). In wheat breeding, the study of agronomic traits plays a crucial role in increasing yield and improving quality (Heidari *et al.* 2024). The results of this study showed that the phenotypic trait coefficient of variation for the tested SHW plants ranged from 1.63% to 22.02% (Table 2). Spike length can reflect the production potential of individual plants to a certain extent. The average spike length of the tested SHW in this study was 12.99 cm, which was 3.33 cm longer than that of Gaoyuan 448 (Tables 2, 3), a major spring wheat variety in Qinghai, and 3.47 cm longer than the results reported by Xu *et al.* (2021). The average plant height of the tested materials was 127.41 cm (Table 2), which differed from the findings of Song *et al.* (2022) and Wang *et al.* (2021). A certain negative correlation exists between plant height and lodging resistance (Navabi *et al.* 2006, Shen *et al.* 2018). Therefore, in subsequent breeding efforts, it is advisable to consider hybridization with shorter-statured wheat cultivars to enhance lodging resistance. Additionally, the significantly longer spike length observed in this study suggests its potential as an excellent parental material for improving spike length in common wheat. The average thousand-grain weight in this study was 39.88 g. However, studies have shown that the performance of SHW in terms of plant height, spike length, and the number of spikelets varies greatly under different ecological conditions. The study by Gao *et al.* (2013) indicated that the average plant height, spike length, and number of spikelets in the SHW population at the Sichuan site were 5.60–7.60 cm, 3.40–3.70 cm, and 4.00–4.10 higher, respectively, than those at the Qinghai site. However, the thousand-grain weight at the Qinghai site was 6.20–28.30 g greater than that at the Sichuan site.

Cluster analysis is widely used in studies on the genetic diversity of wheat germplasm resources and is an effective method for evaluating breeding populations with ideal traits. The results of this study showed that the tested materials could be divided into eight groups, and there were certain differences in traits among different groups. Among them, the materials in cluster VI performed excellently in yield-related traits such as grain area and spike length, and had potential as excellent parental lines for yield traits. The materials in cluster V performed outstandingly in quality traits such as crude protein, gluten, and sedimentation value, especially in crude protein content, and were suitable for use as parental lines with excellent quality traits. At the same time, the results of this study showed that the clustering results based on trait performance were not directly related to the hybrid combination sources of SHW. This may be because the process of creating SHW through distant hybridization between *Triticum turgidum* and *Aegilops tauschii* itself brought about significant trait variation. Therefore, when creating new excellent SHW germplasm, it is not advisable to limit the selection of parents to a few materials with excellent traits. Instead, consideration should be given to expanding the range of parent selection, using more *T.* and *Ae.* as parents, attempting to configure more hybrid combinations, and further enrich the genetic diversity of the entire SHW group, in order to screen and identify new SHW germplasm with more excellent traits.

In this study, we employed grey relational analysis to comprehensively evaluate the agronomic traits of 49 SHW lines. Gray relational analysis allows for a comprehensive quantitative evaluation of multiple traits, making the assessment of the superiority and inferiority of each variety more comprehensive and reliable. Through this method, the superiority and inferiority of the tested materials can be comprehensively judged, avoiding the one-sidedness of traditional methods that rely solely on a single indicator. This provides a more reliable basis for screening and promoting excellent germplasm suitable for the region (Han *et al.* 2021). The results of this study showed that spike length had the highest weight, at 10.08%, followed by the number of spikelets at 9.30% (Table 4). The top 5 germplasms with higher comprehensive evaluations were ABD-SHW-15, ABD-SHW-6, ABD-SHW-5, ABD-SHW-14 and ABD-SHW-38. These lines provide an important germplasm foundation for the development of new wheat cultivars in this region and other high-altitude areas with similar environmental conditions.

However, we also recognize several limitations associated with this approach. Specifically, the current form of grey relational analysis does not allow for flexible adjustment of trait weights according to specific breeding goals or ecological requirements, which limits its precision in guiding actual breeding decisions. In addition, certain traits, such as greater plant height, do not always equate to better performance and should be optimized based on specific regional agronomic needs. Therefore, while this method played a useful role in screening superior germplasm in our study, its outcomes should be interpreted in conjunction with specific breeding objectives and environmental conditions to ensure more robust decision-making. Future work will focus on incorporating additional key agronomic traits and developing weighted evaluation models to enhance the accuracy and applicability of selection strategies.

## Author Contribution Statement

S.Y.: conceptualization, methodology, investigation, formal analysis, writing original draft and editing. J.S.: writing original draft, methodology, investigation, review. F.Y., C.Z. and C.L.: methodology, investigation, writing—review and editing. X.L., M.S. and Q.W.: conceptualization, investigation. R.L.: conceptualization, supervision, writing review. D.L.: methodology, investigation, formal analysis. S.N.: conceptualization, supervision, writing review. L.Z.: provide experimental materials, supervision. H.Z.: formal analysis, supervision. Y.S.: conceptualization, methodology, formal analysis. W.C.: conceptualization, methodology, formal analysis, supervision, writing original draft, writing review and editing, funding acquisition.

## Figures and Tables

**Table 1.** Information on synthetic hexaploid wheat

Germplasm ID	Maternal parent	Origin	paternal parent	Origin	Germplasm ID	Maternal parent	Origin	paternal parent	Origin
ABD-SHW-1	AS313	China	AS60	Iran	ABD-SHW-26	AS2231-2	China	AS77	China
ABD-SHW-2	AS2380	China	AS95		ABD-SHW-27	AS2238	China	AS77	China
ABD-SHW-3	AS2240	China	AS84	Unknown	ABD-SHW-28	AS2291	China	AS2404	Unknown
ABD-SHW-4	AS2255	China	AS93	Unknown	ABD-SHW-29	AS2285	China	AS77	China
ABD-SHW-5	AS2308	China	AS81	China	ABD-SHW-30	PI94675	Georgia	AS2405	Unknown
ABD-SHW-6	AS2310	China	AS60	Iran	ABD-SHW-31	PI113961	Georgia	AS2404	Unknown
ABD-SHW-7	AS2313	China	AS2388	Iran	ABD-SHW-32	PI352335	United States of America	AS2386	Iran
ABD-SHW-8	AS2326	China	AS2388	Iran	ABD-SHW-33	PI352358	France	AS65	Former Soviet Union
ABD-SHW-9	AS2380	China	AS77	China	ABD-SHW-34	PI352369	Czech Republic	AS60	Iran
ABD-SHW-10	AS2382	China	AS2388	Iran	ABD-SHW-35	PI355465	Belgium	AS2405	Unknown
ABD-SHW-11	PI184543	Portugal	AS2386	Iran	ABD-SHW-36	PI355476	Belgium	AS2404	Unknown
ABD-SHW-12	AS2255	China	AS60	Iran	ABD-SHW-37	PI377655	Former Yugoslavia	AS2399	Unknown
ABD-SHW-13	AS313	China	AS60	Iran	ABD-SHW-38	PI377655	Former Yugoslavia	AS2386	Iran
ABD-SHW-14	AS285	Germany	AS60	Iran	ABD-SHW-39	PI211691	Turkey	AS2386	Iran
ABD-SHW-15	AS286	France	AS60	Iran	ABD-SHW-40	PI532136	Egypt	AS65	Former Soviet Union
ABD-SHW-16	Langdon	Unknown	AS60	Iran	ABD-SHW-41	AS286	France	AS60	Iran
ABD-SHW-17	AS2255	China	AS2395	Unknown	ABD-SHW-42	AS2255	China	AS60	Iran
ABD-SHW-18	AS2255	China	AS2393	Iran	ABD-SHW-43	AS2255	China	AS60	Iran
ABD-SHW-19	AS286	France	AS2386	Iran	ABD-SHW-44	Langdon	Unknown	AS60	Iran
ABD-SHW-20	AS286	France	AS2407	Unknown	ABD-SHW-45	AS2310	China	AS60	Iran
ABD-SHW-21	Langdon	Unknown	AS65	Former Soviet Union	ABD-SHW-46	AS2308	China	AS81	China
ABD-SHW-22	Langdon	Unknown	AS77	China	ABD-SHW-47	AS313	China	AS77	China
ABD-SHW-23	Langdon	Unknown	AS2399	Unknown	ABD-SHW-48	AS2231-2	China	AS77	China
ABD-SHW-24	Langdon	Unknown	AS2404	Unknown	ABD-SHW-49	AS286	France	AS2386	Iran
ABD-SHW-25	Langdon	Unknown	AS2407	Unknown					

**Table 2.** Changes in important agronomic traits of synthetic hexaploid wheat from 2022 to 2024

.	Average			Min			Max			CV		
Traits	2022	2023	2024	2022	2023	2024	2022	2023	2024	2022	2023	2024
CP (%)	21.43	20.71	19.68	19.09	18.74	17.06	24.57	24.79	23.81	6.14	6.50	6.85
Gluten (%)	43.24	42.00	40.00	37.85	38.08	35.05	48.67	49.71	45.31	6.51	6.40	6.38
WA (%)	58.52	59.54	61.53	53.08	55.39	51.78	64.59	63.22	65.29	4.33	3.05	4.27
SED (mL)	46.70	47.66	49.16	32.77	30.34	35.22	66.05	61.87	65.67	15.71	16.71	15.09
ST (min)	12.01	11.39	12.58	8.06	7.47	6.35	15.59	15.91	18.00	13.88	17.18	19.64
FT (min)	4.62	4.65	4.96	3.63	3.14	3.40	5.84	5.95	6.81	11.85	14.22	17.34
BD (g L^–1^)	771.17	771.66	778.43	741.33	741.92	738.29	797.66	806.87	808.38	1.71	1.90	1.63
MC (%)	7.64	8.98	10.18	6.77	7.87	9.47	8.58	9.74	10.70	6.09	4.15	3.26
HI (%)	51.46	61.75	64.91	41.48	52.41	55.03	67.97	71.95	73.97	10.93	7.92	6.99
1000-GY (g)	33.23	40.48	45.94	18.53	24.31	27.63	46.00	55.66	58.60	21.33	16.90	14.94
GA (mm^2^)	20.16	20.64	16.81	17.28	12.02	12.00	23.32	25.57	20.37	8.09	13.08	12.32
GL (mm)	8.03	8.18	7.53	7.17	6.49	6.36	8.98	9.10	8.49	4.91	6.03	5.94
GW (mm)	2.98	3.05	3.17	2.62	2.53	2.55	3.38	3.50	3.52	6.47	8.19	7.48
PH (cm)	99.82	136.62	145.78	54.50	101.35	118.50	130.00	154.27	165.67	18.08	7.67	7.00
SL (cm)	13.23	12.66	13.09	8.20	9.85	9.67	19.20	16.17	17.67	17.91	11.74	11.98
SN	15.45	15.60	17.24	11.00	11.00	12.00	20.00	20.67	20.67	15.69	11.52	11.67
SNL (cm)	39.53	51.63	51.65	22.00	32.53	38.67	56.00	73.37	68.00	22.02	14.77	12.10

**Table 3.** Important agronomic traits changes of different germplasm resources in 2022–2024

Germplasm ID	PH (cm)	SL (cm)	SN	SNL (cm)	TGW (g)	GA (mm^2^)	GL (mm)	GW (mm)	CP (%)	Gluten (%)	WA (%)	SED (mL)	ST (min)	FT (min)	BD (g L^–1^)	MC (%)	HI (%)
SHW-ABD-1	*129.89^a^*	*13.43^abcdef^*	*16.00^abcdef^*	*50.17^a^*	*41.11^abc^*	*20.41^ab^*	*7.99^abcd^*	*3.24^abc^*	*19.41^e^*	*39.14^f^*	*60.03^abcd^*	*52.42^abcdef^*	*10.05^fg^*	*4.19^hij^*	*784.48^abcdef^*	*8.97^a^*	*65.37^a^*
SHW-ABD-2	*126.06^a^*	*13.68^abcdef^*	*15.43^abcdef^*	*49.06^a^*	*40.01^abc^*	*20.23^ab^*	*7.92^abcd^*	*3.19^abc^*	*19.61^def^*	*39.49^f^*	*59.41^abcd^*	*49.17^abcdefgh^*	*10.48^fg^*	*4.23^hij^*	*781.44^abcdefgh^*	*8.54^a^*	*56.74^a^*
SHW-ABD-3	*121.54^a^*	*12.47^abcdef^*	*15.89^abcdef^*	*47.92^a^*	*39.68^abc^*	*20.34^ab^*	*8.15^abcd^*	*3.17^abcd^*	*20.26^abcdef^*	*40.86^bcdef^*	*58.39^abcd^*	*53.64^abcd^*	*11.80^bcdefg^*	*4.83^abcdefghij^*	*792.15^abcd^*	*8.52^a^*	*57.11^a^*
SHW-ABD-4	*132.32^a^*	*13.19^abcdef^*	*16.00^abcdef^*	*51.28^a^*	*41.16^abc^*	*20.40^ab^*	*8.06^abcd^*	*3.17^abcd^*	*21.44^abcdef^*	*40.22^cdef^*	*55.53^cd^*	*50.15^abcdefgh^*	*10.61^efg^*	*4.36^efghij^*	*789.42^abcdefgh^*	*8.46^a^*	*60.56^a^*
SHW-ABD-5	*135.18^a^*	*14.43^abcd^*	*14.78^bcdef^*	*49.49^a^*	*32.60^bc^*	*19.42^ab^*	*8.00^abcd^*	*3.07^abcde^*	*23.15^a^*	*45.78^ab^*	*55.07^d^*	*58.27^a^*	*12.38^abcdefg^*	*4.18^hij^*	*781.69^a^*	*8.42^a^*	*59.76^a^*
SHW-ABD-6	*139.61^a^*	*13.34^abcdef^*	*17.11^abcde^*	*53.28^a^*	*43.28^abc^*	*20.84^ab^*	*8.13^abcd^*	*3.22^abc^*	*19.7^abc^*	*45.44^abcd^*	*59.37^abcd^*	*58.40^abc^*	*10.12^fg^*	*4.59^cdefghij^*	*771.25^abc^*	*8.48^a^*	*59.56^a^*
SHW-ABD-7	*130.49^a^*	*13.04^abcdef^*	*15.00^abcdef^*	*51.06^a^*	*38.72^abc^*	*19.13^ab^*	*7.67^abcd^*	*3.09^abcde^*	*19.70^cdef^*	*39.74^f^*	*60.83^ab^*	*55.01^abcdefgh^*	*11.54^cdefg^*	*3.95^j^*	*777.81^abcdefgh^*	*8.73^a^*	*56.81^a^*
SHW-ABD-8	*138.87^a^*	*12.14^abcdef^*	*17.11^abcde^*	*52.78^a^*	*36.32^abc^*	*18.12^ab^*	*7.30^bcd^*	*3.10^abcde^*	*19.44^def^*	*39.98^def^*	*61.36^ab^*	*50.05^abc^*	*15.40^ab^*	*5.87^a^*	*797.03^abc^*	*9.15^a^*	*59.73^a^*
SHW-ABD-9	*118.39^a^*	*13.63^abcdef^*	*16.00^abcdef^*	*43.42^a^*	*36.50^abc^*	*19.62^ab^*	*7.86^abcd^*	*3.15^abcd^*	*20.30^abcdef^*	*40.85^bcdef^*	*61.15^ab^*	*55.18^abcdefgh^*	*11.23^defg^*	*3.85^j^*	*774.04^abcdefgh^*	*8.74^a^*	*57.30^a^*
SHW-ABD-10	*134.84^a^*	*12.80^abcdef^*	*16.22^abcdef^*	*44.03^a^*	*30.05^bc^*	*18.02^ab^*	*7.62^abcd^*	*3.01^abcde^*	*19.71^cdef^*	*40.00^def^*	*60.04^abcd^*	*50.08^abcdef^*	*10.73^efg^*	*3.82^j^*	*774.41^abcdef^*	*8.77^a^*	*54.60^a^*
SHW-ABD-11	*117.52^a^*	*12.17^abcdef^*	*15.56^abcdef^*	*44.73^a^*	*43.64^abc^*	*20.50^ab^*	*8.24^abc^*	*3.11^abcde^*	*20.36^abcdef^*	*38.89^f^*	*58.70^abcd^*	*51.81^abcde^*	*12.19^bcdefg^*	*4.49^defghij^*	*780.64^abcde^*	*8.99^a^*	*55.95^a^*
SHW-ABD-12	*129.39^a^*	*13.60^abcdef^*	*17.33^abcde^*	*50.04^a^*	*41.02^abc^*	*20.71^ab^*	*8.23^abc^*	*3.20^abc^*	*20.17^bcdef^*	*40.74^bcdef^*	*58.57^abcd^*	*52.72^ab^*	*11.03^defg^*	*4.16^ij^*	*780.76^ab^*	*8.96^a^*	*58.31^a^*
SHW-ABD-13	*108.36^a^*	*12.98^abcdef^*	*15.11^abcdef^*	*39.04^a^*	*39.64^abc^*	*22.29^a^*	*8.49^a^*	*3.29^ab^*	*20.93^abcdef^*	*41.91^abcdef^*	*59.54^abcd^*	*56.91^abc^*	*10.60^efg^*	*4.07^j^*	*775.54^abc^*	*8.51^a^*	*55.13^a^*
SHW-ABD-14	*117.81^a^*	*12.27^abcdef^*	*17.33^abcde^*	*50.10^a^*	*29.40^c^*	*17.23^ab^*	*7.64^abcd^*	*2.82^cde^*	*22.43^abcd^*	*45.43^abcd^*	*58.28^abcd^*	*56.46^abcdefghi^*	*11.25^defg^*	*4.83^abcdefghij^*	*772.47^abcdefghi^*	*8.87^a^*	*56.93^a^*
SHW-ABD-15	*127.63^a^*	*15.27^a^*	*19.00^ab^*	*50.61^a^*	*33.46^abc^*	*16.88^ab^*	*7.80^abcd^*	*2.69^e^*	*22.40^abcde^*	*45.57^abc^*	*57.56^abcd^*	*48.23^abcdefghi^*	*13.02^abcdef^*	*5.47^abcdef^*	*761.25^abcdefghi^*	*9.08^a^*	*56.49^a^*
SHW-ABD-16	*104.08^a^*	*14.14^abcde^*	*15.56^abcdef^*	*47.87^a^*	*37.38^abc^*	*18.65^ab^*	*7.91^abcd^*	*2.93^bcde^*	*20.64^abcdef^*	*42.06^abcdef^*	*60.85^ab^*	*46.35^bcdefghij^*	*11.83^bcdefg^*	*4.84^abcdefghij^*	*765.50^bcdefghij^*	*8.98^a^*	*61.09^a^*
SHW-ABD-17	*107.69^a^*	*13.13^abcdef^*	*17.00^abcde^*	*45.34^a^*	*38.46^abc^*	*19.58^ab^*	*7.85^abcd^*	*3.15^abcd^*	*19.84^bcdef^*	*40.42^bcdef^*	*60.20^abc^*	*45.10^abcd^*	*10.96^efg^*	*4.53^cdefghij^*	*774.46^abcd^*	*9.10^a^*	*59.94^a^*
SHW-ABD-18	*105.12^a^*	*12.71^abcdef^*	*13.89^def^*	*42.28^a^*	*40.28^abc^*	*18.15^ab^*	*7.61^abcd^*	*3.12^abcde^*	*21.21^abcdef^*	*43.39^abcdef^*	*59.39^abcd^*	*53.66^hij^*	*12.03^bcdefg^*	*4.72^bcdefghij^*	*776.56^hij^*	*8.98^a^*	*57.65^a^*
SHW-ABD-19	*128.92^a^*	*11.09^def^*	*15.89^abcdef^*	*45.69^a^*	*40.46^abc^*	*18.57^ab^*	*7.88^abcd^*	*3.10^abcde^*	*21.21^abcdef^*	*41.23^bcdef^*	*61.52^ab^*	*37.61^abcdefghi^*	*11.40^cdefg^*	*4.97^abcdefghij^*	*781.11^abcdefghi^*	*8.76^a^*	*67.44^a^*
SHW-ABD-20	*126.54^a^*	*13.49^abcdef^*	*14.89^abcdef^*	*50.47^a^*	*37.38^abc^*	*17.17^ab^*	*7.63^abcd^*	*2.81^cde^*	*20.89^abcdef^*	*42.87^abcdef^*	*60.29^abc^*	*46.16^abcdefg^*	*13.14^abcdef^*	*5.42^abcdefg^*	*771.65^abcdefg^*	*9.13^a^*	*59.61^a^*
SHW-ABD-21	*122.33^a^*	*10.79^ef^*	*15.78^abcdef^*	*48.72^a^*	*41.38^abc^*	*19.58^ab^*	*8.08^abcd^*	*3.08^abcde^*	*21.35^abcdef^*	*43.42^abcdef^*	*59.33^abcd^*	*51.28^abcdefghi^*	*13.07^abcdef^*	*5.25^abcdefghi^*	*781.38^abcdefghi^*	*9.13^a^*	*62.13^a^*
SHW-ABD-22	*133.89^a^*	*12.69^abcdef^*	*16.44^abcdef^*	*53.56^a^*	*44.38^abc^*	*19.15^ab^*	*7.89^abcd^*	*3.05^abcde^*	*20.38^abcdef^*	*42.54^abcdef^*	*62.50^a^*	*47.58^defghij^*	*12.19^bcdefg^*	*4.82^abcdefghij^*	*763.56^defghij^*	*9.37^a^*	*66.38^a^*
SHW-ABD-23	*131.18^a^*	*13.21^abcdef^*	*16.67^abcdef^*	*49.36^a^*	*42.91^abc^*	*18.95^ab^*	*7.86^abcd^*	*3.02^abcde^*	*20.11^bcdef^*	*40.66^bcdef^*	*62.58^a^*	*41.64^efghij^*	*12.63^abcdefg^*	*4.65^bcdefghij^*	*758.17^efghij^*	*8.94^a^*	*60.75^a^*
SHW-ABD-24	*132.64^a^*	*10.89^def^*	*13.78^ef^*	*55.62^a^*	*41.88^abc^*	*15.60^b^*	*7.11^d^*	*2.84^cde^*	*20.06^bcdef^*	*40.27^cdef^*	*60.73^ab^*	*39.95^ij^*	*13.28^abcdef^*	*4.61^cdefghij^*	*761.57^ij^*	*9.02^a^*	*58.55^a^*
SHW-ABD-25	*135.89^a^*	*12.22^abcdef^*	*14.11^def^*	*44.72^a^*	*34.91^abc^*	*18.09^ab^*	*7.52^abcd^*	*2.88^bcde^*	*20.17^bcdef^*	*41.26^bcdef^*	*58.58^abcd^*	*35.77^j^*	*13.43^abcdef^*	*4.60^cdefghij^*	*758.58^j^*	*9.06^a^*	*56.66^a^*
SHW-ABD-26	*126.97^a^*	*12.21^abcdef^*	*19.11^abcdef^*	*36.23^a^*	*38.99^abc^*	*18.40^ab^*	*7.47^abcd^*	*3.12^abcde^*	*19.64^cdef^*	*40.38^bcdef^*	*61.74^ab^*	*33.73^abcdefghi^*	*10.75^efg^*	*4.28^ghij^*	*741.90^abcdefghi^*	*9.10^a^*	*60.22^a^*
SHW-ABD-27	*131.83^a^*	*13.23^abcdef^*	*15.56^abcdef^*	*50.61^a^*	*44.64^abc^*	*20.42^ab^*	*7.93^abcd^*	*3.25^abc^*	*20.13^bcdef^*	*39.50^f^*	*60.12^abc^*	*48.20^cdefghij^*	*12.33^bcdefg^*	*4.59^cdefghij^*	*765.99^cdefghij^*	*8.94^a^*	*61.16^a^*
SHW-ABD-28	*140.54^a^*	*14.14^abcde^*	*15.56^bcdef^*	*48.20^a^*	*51.78^a^*	*21.30^ab^*	*8.01^abcd^*	*3.42^a^*	*19.33^f^*	*39.73^f^*	*62.28^a^*	*43.78^abcdefghij^*	*11.61^cdefg^*	*4.36^efghij^*	*771.80^abcdefghij^*	*8.80^a^*	*57.65^a^*
SHW-ABD-29	*120.38^a^*	*12.98^abcdef^*	*14.78^f^*	*40.40^a^*	*35.00^abc^*	*19.45^ab^*	*7.90^abcd^*	*3.11^abcde^*	*20.04^bcdef^*	*40.70^bcdef^*	*62.56^a^*	*45.80^bcdefghij^*	*9.19^g^*	*4.07^j^*	*756.74^bcdefghij^*	*8.93^a^*	*58.15^a^*
SHW-ABD-30	*124.67^a^*	*10.37^f^*	*12.67^cdef^*	*46.89^a^*	*39.27^abc^*	*19.42^ab^*	*7.84^abcd^*	*3.10^abcde^*	*19.80^bcdef^*	*40.27^cdef^*	*60.07^abcd^*	*44.91^cdefghij^*	*10.88^efg^*	*4.54^cdefghij^*	*773.90^cdefghij^*	*8.97^a^*	*56.39^a^*
SHW-ABD-31	*123.52^a^*	*12.03^abcdef^*	*14.67^cdef^*	*39.38^a^*	*36.20^abc^*	*19.17^ab^*	*8.25^abc^*	*3.01^abcde^*	*20.05^bcdef^*	*46.84^a^*	*62.17^a^*	*43.82^abcdefgh^*	*12.11^bcdefg^*	*5.33^abcdefgh^*	*769.09^abcdefgh^*	*9.31^a^*	*62.69^a^*
SHW-ABD-32	*124.73^a^*	*14.13^abcde^*	*14.67^abcde^*	*40.84^a^*	*40.96^abc^*	*18.55^ab^*	*8.17^abcd^*	*2.88^bcde^*	*22.77^ab^*	*43.52^abcdef^*	*60.16^abc^*	*48.83^cdefghij^*	*12.67^abcdefg^*	*5.45^abcdef^*	*761.82^cdefghij^*	*8.74^a^*	*57.60^a^*
SHW-ABD-33	*126.51^a^*	*13.84^abcdef^*	*17.67^abcde^*	*47.91^a^*	*32.73^bc^*	*18.40^ab^*	*8.24^abc^*	*2.90^bcde^*	*20.92^abcdef^*	*42.99^abcdef^*	*59.40^abcd^*	*43.96^abcdefghi^*	*13.07^abcdef^*	*5.65^abc^*	*767.43^abcdefghi^*	*9.07^a^*	*56.48^a^*
SHW-ABD-34	*132.53^a^*	*14.22^abcde^*	*17.44^abcdef^*	*45.24^a^*	*40.94^abc^*	*19.97^ab^*	*8.25^abc^*	*3.11^abcde^*	*20.91^abcdef^*	*42.89^abcdef^*	*61.78^ab^*	*46.26^abcdefgh^*	*12.48^abcdefg^*	*5.42^abcdefg^*	*772.57^abcdefgh^*	*8.92^a^*	*63.80^a^*
SHW-ABD-35	*131.41^a^*	*13.43^abcdef^*	*16.89^abcde^*	*43.62^a^*	*34.78^abc^*	*17.19^ab^*	*7.53^abcd^*	*2.89^bcde^*	*21.40^abcdef^*	*45.32^abcde^*	*59.49^abcd^*	*48.82^abcdefghi^*	*15.00^abc^*	*5.76^ab^*	*771.90^abcdefghi^*	*9.49^a^*	*59.82^a^*
SHW-ABD-36	*127.12^a^*	*15.03^ab^*	*17.56^cdef^*	*46.89^a^*	*39.53^abc^*	*18.46^ab^*	*7.82^abcd^*	*3.00^abcde^*	*21.63^abcdef^*	*43.59^abcdef^*	*60.14^abc^*	*47.55^fghij^*	*9.05^g^*	*4.76^abcdefghij^*	*768.60^fghij^*	*8.94^a^*	*59.05^a^*
SHW-ABD-37	*121.49^a^*	*11.43^bcdef^*	*14.67^cdef^*	*44.12^a^*	*37.93^abc^*	*15.81^b^*	*7.23^cd^*	*2.82^cde^*	*21.01^abcdef^*	*43.90^abcdef^*	*61.62^ab^*	*39.87^abcdef^*	*12.94^abcdef^*	*5.60^abcd^*	*766.15^abcdef^*	*9.14^a^*	*60.10^a^*
SHW-ABD-38	*136.28^a^*	*11.59^bcdef^*	*14.56^abc^*	*53.87^a^*	*42.21^abc^*	*18.59^ab^*	*8.20^abc^*	*2.94^bcde^*	*21.19^abcdef^*	*44.10^abcdef^*	*57.89^abcd^*	*52.20^abcdefghi^*	*14.70^abcd^*	*5.76^ab^*	*783.72^abcdefghi^*	*9.39^a^*	*60.15^a^*
SHW-ABD-39	*141.86^a^*	*13.73^abcdef^*	*18.67^abcdef^*	*55.17^a^*	*41.48^abc^*	*17.94^ab^*	*8.03^abcd^*	*2.84^cde^*	*21.09^abcdef^*	*39.88^ef^*	*59.39^abcd^*	*47.73^bcdefghij^*	*12.68^abcdefg^*	*4.51^cdefghij^*	*776.27^bcdefghij^*	*8.58^a^*	*59.68^a^*
SHW-ABD-40	*122.51^a^*	*11.28^cdef^*	*15.22^abcde^*	*37.53^a^*	*48.32^ab^*	*20.87^ab^*	*8.33^ab^*	*3.15^abcd^*	*19.68^cdef^*	*39.74^f^*	*58.18^abcd^*	*45.06^abcdef^*	*11.33^cdefg^*	*4.36^efghij^*	*774.06^abcdef^*	*8.91^a^*	*60.72^a^*
SHW-ABD-41	*128.99^a^*	*14.96^ab^*	*17.44^abcdef^*	*53.39^a^*	*35.45^abc^*	*17.64^ab^*	*7.99^abcd^*	*2.74^de^*	*20.42^abcdef^*	*40.78^bcdef^*	*58.61^abcd^*	*51.86^abcdefgh^*	*15.96^a^*	*5.50^abcde^*	*787.97^abcdefgh^*	*9.20^a^*	*57.88^a^*
SHW-ABD-42	*126.56^a^*	*14.74^abc^*	*15.56^abcde^*	*47.40^a^*	*40.55^abc^*	*20.85^ab^*	*8.33^ab^*	*3.17^abcd^*	*19.63^def^*	*39.56^f^*	*56.70^bcd^*	*48.89^ghij^*	*14.21^abcde^*	*4.67^bcdefghij^*	*790.25^ghij^*	*9.04^a^*	*59.63^a^*
SHW-ABD-43	*128.07^a^*	*13.67^abcdef^*	*17.00^abc^*	*51.84^a^*	*46.34^abc^*	*20.65^ab^*	*8.01^abcd^*	*3.25^abc^*	*19.87^bcdef^*	*41.04^bcdef^*	*57.68^abcd^*	*38.90^bcdefghij^*	*9.88^fg^*	*4.11^ij^*	*777.40^bcdefghij^*	*8.80^a^*	*60.06^a^*
SHW-ABD-44	*138.54^a^*	*13.88^abcdef^*	*18.56^abcdef^*	*52.82^a^*	*44.48^abc^*	*18.38^ab^*	*7.75^abcd^*	*3.06^abcde^*	*20.50^abcdef^*	*42.20^abcdef^*	*60.05^abcd^*	*44.42^abcd^*	*10.45^fg^*	*4.93^abcdefghij^*	*767.14^abcd^*	*8.83^a^*	*60.39^a^*
SHW-ABD-45	*130.59^a^*	*14.81^abc^*	*16.11^abcdef^*	*49.96^a^*	*41.47^abc^*	*20.86^ab^*	*8.14^abcd^*	*3.25^abc^*	*20.94^abcdef^*	*42.73^abcdef^*	*61.07^ab^*	*53.10^abcdefgh^*	*13.06^abcdef^*	*4.89^abcdefghij^*	*771.53^abcdefgh^*	*8.95^a^*	*60.73^a^*
SHW-ABD-46	*117.78^a^*	*13.37^abcdef^*	*15.44^abcd^*	*42.43^a^*	*45.51^abc^*	*21.14^ab^*	*8.26^abc^*	*3.24^abc^*	*20.90^abcdef^*	*42.35^abcdef^*	*59.01^abcd^*	*48.83^abcdefghi^*	*11.29^cdefg^*	*4.49^defghij^*	*781.82^abcdefghi^*	*9.12^a^*	*59.51^a^*
SHW-ABD-47	*129.92^a^*	*13.01^abcdef^*	*18.11^abcde^*	*51.29^a^*	*41.45^abc^*	*20.00^ab^*	*7.86^abcd^*	*3.17^abcd^*	*20.28^abcdef^*	*40.41^bcdef^*	*60.91^ab^*	*46.95^defghij^*	*12.52^abcdefg^*	*4.81^abcdefghij^*	*777.21^defghij^*	*8.86^a^*	*59.48^a^*
SHW-ABD-48	*126.32^a^*	*13.33^abcdef^*	*17.67^abcdef^*	*48.11^a^*	*47.54^abc^*	*20.82^ab^*	*7.86^abcd^*	*3.32^ab^*	*19.36^f^*	*39.49^f^*	*61.22^ab^*	*42.16^bcdefghij^*	*11.87^bcdefg^*	*4.34^fghij^*	*781.45^bcdefghij^*	*9.27^a^*	*60.54^a^*
SHW-ABD-49	*129.72^a^*	*10.96^def^*	*15.33^a^*	*47.83^a^*	*40.70^abc^*	*19.08^ab^*	*7.92^abcd^*	*3.01^abcde^*	*20.40^abcdef^*	*41.44^bcdef^*	*60.98^ab^*	*45.08^a^*	*11.27^defg^*	*4.82^abcdefghij^*	*770.37^a^*	*8.73^a^*	*56.83^a^*
Average	*127.41*	*12.99*	*16.10*	*47.60*	*39.88*	*19.20*	*7.91*	*3.07*	*20.61*	*41.75*	*59.86*	*47.84*	*11.99*	*4.74*	*773.76*	*8.93*	*59.37*
Gaoyuan 448	*99.23*	*9.67*	*17.67*	*37.55*	*41.55*	*15.54*	*5.86*	*3.54*	*14.89*	*29.03*	*60.80*	*---*	*6.10*	*2.95*	*811.90*	*6.08*	*70.60*

**Table 4.** The distribution of weights assigned to different traits

Traits	Weight (%)	Traits	Weight (%)	Traits	Weight (%)
SL	10.08	SNL	7.26	FT	3.58
SN	9.30	ST	7.14	BD	3.18
HI	8.62	Gluten	6.86	GW	1.87
MC	8.41	TGW	6.46	WA	1.57
CP	8.40	GL	4.59	SED	1.29
PH	7.56	GA	3.83		

**Table 5.** Comprehensive evaluation results between different germplasm resources

Germplasm ID	Score	Rank		Germplasm ID	Score	Rank		Germplasm ID	Score	Rank
SHW-ABD-1	50.02	43		SHW-ABD-18	55.61	21		SHW-ABD-35	60.02	6
SHW-ABD-2	50.42	40		SHW-ABD-19	55.45	23		SHW-ABD-36	58.50	10
SHW-ABD-3	53.65	29		SHW-ABD-20	58.75	9		SHW-ABD-37	57.84	12
SHW-ABD-4	56.13	19		SHW-ABD-21	59.28	8		SHW-ABD-38	62.33	5
SHW-ABD-5	63.70	3		SHW-ABD-22	57.45	14		SHW-ABD-39	56.79	18
SHW-ABD-6	64.39	2		SHW-ABD-23	53.36	30		SHW-ABD-40	46.68	48
SHW-ABD-7	51.08	37		SHW-ABD-24	55.19	24		SHW-ABD-41	57.13	16
SHW-ABD-8	54.72	25		SHW-ABD-25	52.19	34		SHW-ABD-42	50.65	39
SHW-ABD-9	50.32	42		SHW-ABD-26	46.54	49		SHW-ABD-43	53.14	31
SHW-ABD-10	48.48	47		SHW-ABD-27	52.77	33		SHW-ABD-44	57.33	15
SHW-ABD-11	50.39	41		SHW-ABD-28	49.92	44		SHW-ABD-45	57.63	13
SHW-ABD-12	52.97	32		SHW-ABD-29	48.90	46		SHW-ABD-46	53.68	28
SHW-ABD-13	51.37	35		SHW-ABD-30	51.23	36		SHW-ABD-47	54.51	26
SHW-ABD-14	63.18	4		SHW-ABD-31	56.01	20		SHW-ABD-48	49.71	45
SHW-ABD-15	64.53	1		SHW-ABD-32	59.89	7		SHW-ABD-49	54.40	27
SHW-ABD-16	55.50	22		SHW-ABD-33	58.37	11				
SHW-ABD-17	50.85	38		SHW-ABD-34	56.85	17				
